# Partial Ramollissement of the Brain, Accompanied with Remarkable Symptoms

**Published:** 1830-01-01

**Authors:** 


### XXXIII.

### PARTIAL RAMOLLISSEMENT OF THE BRAIN, ACCOMPANIED WITH REMARKABLE SYMPTOMS.

The following very interesting cases are taken from the second edition of Dr. Abercrombie's excellent work on the Diseases of the Brain and Spinal Cord. The phenomena do not square with the commonly received notions respecting affections of the head, but facts are more valuable than prejudices, and the quarter from which the present emanate is a sufficient guarantee for their accuracy and truth.

*Case* 1. "A gentleman, aged 26, of a plethoric habit, had suffered occasionally for two or three years from headach and vertigo, which were always relieved by depletion. On 12th April, 1827, while walking out, he was seized with confusion and giddiness, embarrassed speech, and a considerable degree of paralysis of the right leg. He was rather pale; his pulse was 70 and soft; and he did not complain of any headach. The usual treatment was adopted with activity by Dr. Combe of Leith, without much relief. On the contrary, after several days he began to complain of acute headach, accompanied by vomiting and hiccup; and the other symptoms continued nearly as before,—his speech being laboured and slow, and his memory very defective. After some weeks those symptoms subsided, so that he was able to walk out; but the headach continued with frequent vomiting. The pain was chiefly referred to the left side of the head, sometimes to the occiput, and there was occasional numbness of the right arm. When I saw him, along with Dr. Combe and Dr. Kelly in July, his chief complaint was of frequent and irregular attacks of vomiting, occurring daily, or repeatedly during the day. It came on very suddenly, without previous nausea, and he was often awakened in the night by the sudden attack of vomiting. He had now a pale sickly look; there was no paralytic affection, and little complaint of headach; though he still had occasional uneasiness in the head, sometimes referred to one part of it and sometimes to another. When he did refer it to a particular part as the principal seat of the pain, it was either the left temple or the occiput. But the headach at this time was slight and transient, and the symptoms in the stomach were so much the more prominent, that it was a matter of much doubt whether there was now any fixed disease in the head. The vomiting was much relieved by the oxyd of bismuth, so that he was free from it for several days. But it soon returned and went on as before, with increasing debility, great listlessness, and bad appetite; pulse little affected. He had now a peculiar unsteadiness of his limbs, so that on first getting up into a standing posture, he staggered very much and required some time and attention to steady himself. When he had accomplished this he walked with tolerable firmness. The symptoms went on in this manner till the 27th of October, when he was suddenly seized with violent and continued convulsion, and died in nine hours.

"*Inspection.*—In the substance of the middle lobe of the left hemisphere of the brain, about the level of the lateral ventricle, there was a portion in a state of complete ramollissement, about an inch and a half in length, and an inch in its other dimensions, and the neighbouring parts appeared unusually vascular. The tuber annulare and Pons Varolii were softer than usual, but otherwise healthy. No other morbid appearance could be discovered in the head, and all the other viscera were healthy.

"It is unnecessary to point out the very remarkable features of this case. The sudden attack so closely resembling the ordinary paralytic attack, must have been connected with the commencement of the inflammatory stage. The remarkable symptoms in the stomach in the farther progress of the disease, and the mode of its termination, make it altogether a case of great value in the pathology of this remarkable affection."

The connexion between disorder of the stomach and affection of the brain, in other words the sympathy between the organs, though frequently difficult to explain is still sufficiently established. Every body knows that a blow upon the head produces vomiting, and an attack of apoplexy is often preceded by nausea and a disposition to sickness. But it is in chronic diseases of the encephalon that we witness this mysterious sympathy most curiously displayed, and cases have occurred in which sickness or other disorder of the stomach has absorbed the whole attention of the medical practitioner and patient himself, whilst the real mischief was marching on within the head to a fatal termination. In the foregoing case it is hinted that a similar mistake was fallen into, and in a former number of this Journal* a very instructive instance of the same kind is narrated. The case in question was that of a woman whose prominent symptom was obstinate sickness at stomach which no medicines could arrest, and which at length proved the immediate cause of her death. On dissection the stomach and bowels were found to be perfectly sound but there was a small soft tumour in each posterior lobe of the cerebrum, and a similar mass in a suppurated state in the left hemisphere of the cerebellum. For the particulars of the case we refer to our more detailed account of it, and we think that it forms a good precedent to that of Dr. Abercrombie.

In the following the same morbid appearances as those in Dr. A.'s first case are accompanied with a very different train of symptoms. It is a significant hint to those enthusiastic gentlemen who put their undivided trust in morbid anatomy, and swear by the scalpel with more devotion than the Turk by the beard of the Prophet, or the Persian by the sun.

*Case* 2. "A gentleman, aged 38, during two years before his death had suffered several epileptic attacks, from which, however, he had always speedily recovered. On the morning of 27th

* No. 10, New Series, page 584.

December 1827, he was found in bed speechless and paralytic on the right side. He recovered his speech in the course of the day: the palsy continued in the usual manner, and after some time he began to recover a degree of motion of the parts. When he came to Edinburgh about a month after the attack, he had recovered the use of his leg so far as to be able to walk once or twice across his room with much exertion; his arm was improved in a much less degree; his speech was distinct, but his mouth was considerably distorted, and his mind was somewhat impaired. He now consulted Dr. Thomson, and under the usual treatment he was progressively improving, so that at the end of another month he could walk along the streets to a considerable distance, though with a dragging motion of his leg, and could nearly raise his arm to his head. In the evening of 22d February he went to a supper party, and seemed remarkably well; but departed considerably from the abstemious regimen to which he had been previously restricted. About 8 o'clock on the morning of the 23d he was found in bed in a state of complete insensibility, accompanied by severe and general convulsion, which was strongest in the limbs of the right side. The face was much convulsed, the eyes rolling and insensible, the respiration laborious and convulsive. Blood-letting and the other usual means were actively employed without any relief. The convulsion continued unabated in the state now described, when I saw him at eleven, and he died at two.

"*Inspection.*—The brain externally was healthy, except some old adhesion of the membranes near the posterior part of the falx, and very trifling effusion under the arachnoid. The ventricles contained the usual very small quantity of fluid. On the outer side of the left ventricle, and separated from it by a thin partition of healthy cerebral substance, there was a defined portion in a state of complete and diffluent ramollissement. The portion thus affected was about an inch in depth; about half or 3-4ths of an inch in diameter at the upper part, and became gradually narrower as it descended by the side of the ventricle, until it terminated almost in a point. There was considerable softening of part of the medulla oblongata, and the upper part of the spinal cord. No other vestige of disease could be discovered on the most careful examination."

In connexion with these cases of partial softening of the brain we may mention one of a similar character in some respects, which we lately witnessed at St. George's Hospital.

*Case* 3. Elizabeth Fry, ætatis 40, was admitted into that Institution on the 30th of September last under the care of Dr. Seymour. Her symptoms were pain in the head affecting chiefly the space between the eye-brows and occasionally the occiput—pupils unaffected — sense of smell lost — limbs weaker than they should be, but not hemiplegiac — tongue clean — bowels open — body rather emaciated. The pain in the head was said to have come on five weeks previously after the sudden cessation of the catamenia. Cupping to the back of the neck, the ammoniated tincture of valerian in camphor mixture, the tepid bath, leeches to the forehead, and blisters to the same part and behind the ears, were the measures successively had recourse to. The patient however became worse instead of better, and towards the latter end of October was so weak as to require wine and ammonia. She rallied and on the 26th complained of intense pain between the eye-brows, frequent aberration of intellects, involuntary discharge of fæces; the pulse was regular and natural and there was no hemiplegia. Frictions with the antimonial ointment and opening medicine were prescribed, but at 4 p. m. of the 29th, the patient was discovered in a state of complete coma—loss of power over the muscles of the body—perfect but not loud stertor — dilated pupils — slow struggling pulse, all the symptoms in fact of perfect apoplexy. She was bled to twelve ounces without any other effect than that of increasing the frequency of the pulse, and at 8 p.m. she was bled again to sixteen ounces from the temporal artery. As before, the pulse rose from 50 to 120 after the abstraction of blood, but no amelioration in the symptoms took place and she died in the course of the night.

*Sectio Cadaveris.* The body was emaciated, but not to an extreme degree. In the cranium:—the vessels of the pia mater were not unusually loaded with venous blood, nor was there any effusion beneath the arachnoid; a very considerable quantity of clear yellow serum in the lateral ventricles which appeared to be enlarged.

In the right hemisphere of the brain, partly in its anterior, partly in its middle lobe, and stretching across the fissura sylvii was a curious morbid condition of parts. It was a darkish, almost sloughy looking cavity, partly containing recent coagulum but principally filled with a diffluent substance looking like a mixture of softened cerebral matter and effused blood of ancient, or at all events not very late date. Besides these appearances there were one or two nodules the size of marbles, nearly circular, firm, softened, and as it were, putrescent in their centre, and one of them fibrous on section. The cerebral matter in the immediate neighbourhood of the disease was tinged of a darkish colour, apparently from mere contiguity, as it carried no unusual quantity of red blood nor presented any trace of increased organization; there was no pus—no gangrenous odour. Nothing further of any consequence was discovered in the brain, nor was there any disease in the thoracic or abdominal cavities.

The general opinion amongst those who witnessed the examination was, that of the disease being a specimen of fungus hæmatodes of the brain. Whether such opinion be correct or not, the case is undoubtedly one of much interest, and may not unfairly be united with the two recorded by Dr. Abercrombie of partial ramollissement.

---

XXXIV.

